# The association of *HLA-G* polymorphisms and the synergistic effect of sMICA and sHLA-G with chronic kidney disease and allograft acceptance

**DOI:** 10.1371/journal.pone.0212750

**Published:** 2019-02-22

**Authors:** Vanessa Hauer, Matilde Risti, Bruna L. M. Miranda, José S. da Silva, Ana L. Cidral, Carolina M. Pozzi, Fabiana L. de C. Contieri, Ibrahim A. Sadissou, Eduardo A. Donadi, Danillo G. Augusto, Maria da G. Bicalho

**Affiliations:** 1 Laboratório de Imunogenética e Histocompatibilidade, Departamento de Genética, Universidade Federal do Paraná, Curitiba, Paraná, Brazil; 2 Setor de Transplante Renal, Hospital Universitário Evangélico de Curitiba, Alameda Augusto Stellfeld, Curitiba, Brazil; 3 Laboratório de Biologia Molecular, Departamento de Clínica Médica, Faculdade de Medicina de Ribeirão Preto da Universidade de São Paulo, Ribeirão Preto, São Paulo, Brazil; 4 Laboratório de Genética Molecular Humana, Departamento de Genética, Universidade Federal do Paraná, Curitiba, Paraná, Brazil; 5 Departamento de Ciências Biológicas, Universidade Estadual de Santa Cruz, Ilhéus, Bahia, Brazil; University of Mississippi Medical Center, UNITED STATES

## Abstract

The *HLA-G* and *MICA* genes are stimulated under inflammatory conditions and code for soluble (sMICA and sHLA-G) or membrane-bound molecules that exhibit immunomodulatory properties. It is still unclear whether they would have a synergistic or antagonistic effect on the immunomodulation of the inflammatory response, such as in chronic kidney disease (CKD), contributing to a better prognosis after the kidney transplantation. In this study, we went from genetic to plasma analysis, first evaluating the polymorphism of *MICA*, *NKG2D* and *HLA-G* in a cohort from Southern Brazil, subdivided in a control group of individuals (n = 75), patients with CKD (n = 94), and kidney-transplant (KT) patients (n = 64). *MICA*, *NKG2D* and *HLA-G* genotyping was performed by polymerase chain reaction with specific oligonucleotide probes, Taqman and Sanger sequencing, respectively. Levels of soluble forms of MICA and HLA-G were measured in plasma with ELISA. Case-control analysis showed that the individuals with haplotype HLA-G*01:*01/*UTR-4 have a lower susceptibility to develop chronic kidney disease (OR = 0.480; *p* = 0.032). Concerning the group of kidney-transplant patients, the *HLA-G* genotypes *+3010 GC* (*rs1710*) and *+3142 GC* (*rs1063320*) were associated with higher risk for allograft rejection (OR = 5.357; p = 0.013 and OR = 5.357, p = 0.013, respectively). Nevertheless, the genotype *+3010 GG* (OR = 0.136; *p* = 0.041) was associated with kidney allograft acceptance, suggesting that it is a protection factor for rejection. In addition, the phenotypic analysis revealed higher levels of sHLA-G (*p* = 0.003) and sMICA (*p* < 0.001) in plasma were associated with the development of CKD. For patients who were already under chronic pathological stress and underwent a kidney transplant, a high sMICA (*p* = 0.001) in pre-transplant proved to favor immunomodulation and allograft acceptance. Even so, the association of genetic factors with differential levels of soluble molecules were not evidenced, we displayed a synergistic effect of sMICA and sHLA-G in response to inflammation. This increase was observed in CKD patients, that when undergo transplantation, had this previous amount of immunoregulatory molecules as a positive factor for the allograft acceptance.

## Introduction

Previous studies on the major histocompatibility complex (MHC) genomic region identified genes that are important for immune regulation [1–3]. Among these genes are *HLA-G* (human leukocyte antigen-G) and *MICA* (major histocompatibility complex class I chain-related gene A). The few studies that concomitantly evaluated those genes left questions to be clarified about their functions [4,5]. HLA-G and MICA are highlighted, because they are produced in inflammatory and pathological conditions [5,6], can be expressed on cell membranes and reach distant immunological targets when in the form of soluble isoforms (sHLA-G and sMICA) [7,8].

HLA-G is expressed in regulatory T-cells and endothelial cells [9]; its expression has also been observed in transplanted specimens and associated with better graft survival [10–14]. The immunomodulatory role of HLA-G is performed mainly through interaction with inhibitory receptors, such as the leukocyte Ig-like receptor family B member 1 (LILRB1) and member 2 (LILRB2) [15]. Soluble HLA-G induces regulatory mechanisms, such as apoptosis of CD8^+^T and NK cells, inhibition of B-cell proliferation, differentiation, and Ig secretion [16]. The membrane-bound HLA-G1 and the secreted soluble HLA-G5 are the most widely investigated isoforms [7,8].

Considering the immunomodulatory role of *HLA-G*, its allelic variation may impact its function on immunoregulation [17]. For example, the G*01:*04* allele group has been associated with increased expression levels of sHLA-G in kidney-transplant patients without acute rejection [18]. Still, the 3’-UTR *HLA-G* region (exon 8) has nine polymorphisms with cumulative effect towards differential levels of sHLA-G [14,19].

MICA is expressed in various cells, including the thymic medulla and the gastrointestinal epithelium [6,20–24]. The best-described interaction of MICA occurs with the natural killer group 2 member D ligand (NKG2D). This receptor is expressed in natural killer (NK) cells, γδ Τ-cells, and αβ CD8^+^ T-cell membranes [25].

Functionally, the interaction of NKG2D with the membrane MICA can culminate in cytotoxic activity of NK cells, but this signal can be counteracted by HLA-G interaction with LILRB1 inhibitory signal [4]. However, MICA as a soluble isoform may induce internalization and degradation of NKG2D [20]. This can originate an arrest of CD4+ T cells cycle in the G0-G1 phases, as well as induce proliferation of immunosuppressive NKG2D^+^CD4^+^T cells, able to act in a paracrine way, inducing growth arrest of other T cells, secretion of IL-10 and TGF-β, similarly to the T regulatory type 1 cell cytokine profiles [26].

The number of GCT repetitions at exon 5 generates five different *MICA* alleles (*MICA-A4*, *A5*, *A5*.*1*, *A6*, and *A9*). The *A5*.*1* allele (dbSNP: *rs41293539*) contains five GCT repetitions and one insertion, which leads to a frameshift mutation and generates a truncated protein with a differential expression, cellular localization [27] and was related to a bad outcome in kidney transplantation [28]. The *MICA-129 Val/Met* polymorphism (dbSNP: *rs1051792*) at nucleotide 454 (G>A) leads to a substitution, Val129Met in the α2 domain of the MICA protein [25]. The MICA-129 Val/Met variant can affect NKG2D binding avidity, leading to an alteration in the immune response mediated by NK cells, which also depends on the level of *MICA* expression [29,30]. The *NKG2D* gene has two haploblocks (set of haplotypes), hb1 and hb2. The first haploblock (hb1) can be discriminated in high and low natural cytotoxic activity (*HNK1* and *LNK1*, respectively). *HNK1* (described by the main dbSNP, rs1049174-G) is associated with higher activity of NK cells in the peripheral blood and a lower incidence of cancers originating from epithelial cells [25,31].

The immunomodulatory effects of MICA with NKG2D and HLA-G, together with the scarcity of data on the diversity of genes and their influence on the composition of these soluble molecules, stimulated the development of this study. We hypothesized, that genetic variation in these genes may be associated with differential levels of plasmatic sHLA-G and sMICA. These immunological alterations would be related to the susceptibility of developing chronic kidney disease and with the establishment of the immunological balance or rejection after renal transplantation. Here, we evaluated the impact of *MICA*, *NKG2D* and *HLA-G* genotypes as well as the sMICA and sHLA-G levels, comparing patients with chronic kidney disease with controls and patients who had undergone a kidney transplant and developed rejection with those who did not.

## Methods

### Sample

The variation of *HLA-G*, *MICA* and *NKG2D* was investigated in individuals from the state of Paraná, Southern Brazil. This sample was composed of 169 individuals, divided between a control group of individuals in homeostasis, without chronic kidney disease or other uncontrolled inflammatory disease (Ct, n = 75) and patients in the end stage of chronic kidney disease (CKD, n = 94). Of the CKD, a total of 64 patients (in the end stage of renal disease, with a glomerular filtration rate under 15 mL/min, calculated following the Chronic Kidney Disease Epidemiology Collaboration equation [32]) had undergone kidney transplantation along 2012 and 2013, and had developed rejection (KTR, n = 28), or had not (KTN, n = 36). The information regarding the development or not of kidney allograft-rejection episodes (such as acute cellular rejection, chronic allograft nephropathy, and/or chronic glomerulonephritis) was obtained up to 4 years after transplantation.

The characteristics of the population (Ct, CKD and KT patients) are described in Table 1. All participants gave their signed informed consent before blood collection. The study was approved by the Ethics Committee of the Federal University of Paraná, Brazil and the Evangelical University Hospital of Curitiba, Paraná, Brazil (protocol number: CAAE 53627315.0.0000.0102).

**Table 1:** Demographic characterization of the sample.

Characteristics	Ct (N = 75)	CKD (n = 94)	*p*	KTN (n = 36)	KTR (n = 28)	*p*
**Male**	34.70%	59.60%	0.002^1^	47.20%	75.00%	0.023^1^
**Age**	37.88 years (SD = 14.46)	46.77 years (SD = 13.44)	<0.001^1^	44.92 years (SD = 14.27)	46.32 years (SD = 13.67)	0.692
**Ethnic group** ^2^						
**Mixed race**	10.80%	9.60%	1.000	8.30%	17.90%	0.448
**Asian**	1.40%	2.10%	1.000	2.80%	0.00%	1.000
**Black**	0.00%	6.40%	0.034	2.80%	7.10%	0.577
**Mulatto**	0.00%	2.10%	0.503	0.00%	7.10%	0.187
**White**	87.80%	79.80%	0.211	86.10%	67.90%	0.127
**Diseases**						
**Hypertension**	16.00%	90.30%	<0.001	54.20%	45.80%	0.375
**Diabetes**	2.70%	23.70%	<0.001	19.40%	14.30%	0.743
**Dyslipidemias**	2.70%	16.10%	0.004	11.10%	7.10%	0.688
**Glomerulonephritis**	0.00%	37.60%	<0.001	36.10%	46.80%	0.450
**Hypertensive nephropathy**	0.00%	14.90%	<0.001	44.40%	55.60%	0.488
**Transplant patients**				**KTN (n = 36)**	**KTR (n = 28)**	
**Male donors**				41.70%	50.00%	0.615
**Living donors**				41.70%	25.00%	0.193
**Donor’s age**				40.39 years (SD = 12.29)	48.18 years (SD = 12.07)	0.014^1^
**Previous transplants**				19.40%	14.30%	0.743
**Previous blood transfusion**				50.00%	35.70%	0.313
**Multiparous women** ^3^				42.11%^3^	^3^	0.378^3^
**Detection of DSA**				5.60%	21.40%	0.064
***HLA* mismatches**						
***HLA-A*—0/1/2**			25.00%/41.70%/16.70%		10.70%/42.90%/35.70%	0.141
***HLA-B*—0/1/2**			22.20%/50.00%/11.10%		00.00%/50.00%/39.30%	0.003^1^
***HLA-DRB1*–0/1/2**			25.00%/50.00%/8.30%		21.40%/42.90%/25.00%	0.227
**Unknown**			16.70%		10.7%	0.720
**Time on dialysis**			38 months (SD = 33)		55 months (SD = 51)	0.100
**GFR**			7.58 mL/min (SD = 3.44)		7.11 mL/min (SD = 4.86)	0.662
**Cold ischemia time**^4^			15h:31min (SD = 6h:46min)		14h:44min (SD = 8h:21min)	0.754

Ct: Control group. CKD: Patients with chronic kidney disease. KTN: Kidney-transplant patients with no rejection. KTR: Kidney-transplant patients who developed episodes of rejection. SD: standard deviation. DSA: donor-specific antibody. GFR: Glomerular filtration rate in pre-transplant, calculated following CKD-EPI (Chronic Kidney Disease Epidemiology Collaboration) equation.

^1^Analyses performed with adjustment for sex and age.

^2^Ethnicity information was obtained by self-assessment.

^3^Calculated from the total number of females in the group.

^4^Calculated only for deceased donors. The cold ischemia time for all living donors was a maximum of 1 hour.

### Immunosuppression protocol from kidney-transplant patients

For all KT patients, pre-transplant immunosuppression was based on intravenous corticosteroid administration (methylprednisolone, 500 mg, independently of weight). Patients with increased immunological risk for allograft rejection received induction therapy with anti-thymocyte globulin (ATG). Maintenance therapy was composed of three medications: a corticosteroid (prednisone), a calcineurin inhibitor (tacrolimus), and an antimetabolite agent (mycophenolate mofetil, which was replaced by mycophenolate sodium after discharge).

### *HLA-G*, *MICA* and *NKG2D* genotyping

Blood samples were collected in tubes containing ethylene-diamine-tetra-acetic acid (EDTA), and DNA was extracted from buffy-coat samples, using a salting-out technique [33].

The sequencing of *HLA-G* gene was performed by Sanger method. We used the ABI Prism Big Dye Terminator v3.1 Cycle Sequencing kit (Applied Biosystems, CA, USA), and analyzed the data in ABI Prism SeqScape software version 2.7 (Applied Biosystems). Exons 2, 3 and 4 were amplified and sequenced using the following primers: i) exon 2, F2E:GGGTCGGGCGGGTCTCAA, and R2E: TCCGTGGGGCATGGAGGT, ii) exon 3, F3E:CCCAGACCCTCTACCTGGGAGA, R3E:CTCTCCTTGTGCTAGGCCAGGCTG, iii) exon 4, F4E: CCATGAGAGATGCAAAGTGCT and R4E:TGCTTTCCCTAACAGACATGAT, as adapted from a previous study [34]. All sequences were analyzed based on the official alleles listed in the International Immunogenetics Information System (IMGT) [35].

Amplification of the *HLA-G* 3′-UTR was performed using the following primers: HLA-G8F:TGTGAAACAGCTGCCCTGTGT [36] and HGUT.R1:GTCTTCCATTTA TTTTGTCTCT [33]. PCR was carried out in a final volume of 25 μL, containing one unit of Hot Start Go *Taq* DNA-polymerase (Promega, Madison, WI, USA), 1× of Colorless GoTaq Flexi Buffer (Promega), 0.2 mM of each dNTP, 1.5 mM MgCl_2_, 0.4 pmol of each primer, and 25 ng of genomic DNA. The initial denaturation was carried out at 94°C for 5 min, followed by 32 cycles of 95°C for 45 s, 57°C for 45 s and 72°C for 1 min, with a final extension step at 72°C for 7 min. This protocol, adapted from Castelli [37], was used for all samples.

Enzymatic purification of amplicons was performed and exon 8 was sequenced using the reverse primer to prevent sequence overlap in heterozygous genotypes due to the 14-bp indel (+2960, dbSNP: *rs371194629*). Including the 14-bp *Ins/del*, a total of 17 sites described in Table 2 were analyzed and individually annotated in the amplicon.

**Table 2:** The 17 sites of the *HLA-G* 3’-UTR region analyzed in this study.

**rs371194629**	14-bp *Del*	14-bp *Ins*	+2960
**rs567747015**	*C*	*T*	+3001
**rs1707**	*C*	*T*	+3003
**rs1710**	*G*	*C*	+3010
**rs17179101**	*C*	*A*	+3027
**rs146339774**	*G*	*C*	+3032
**rs17179108**	*C*	*T*	+3035
**No SNPidNo SNPid**^1^	*A*	*T*	+3044
**No SNPidNo SNPid**^1^	*C*	*T*	+3052
**rs180827037**	*G*	*T*	+3092
**No SNPidNo SNPid**^1^	*C*	*G*	+3107
**No SNPidNo SNPid**^1^	*G*	*A*	+3111
**rs138249160**	*T*	*C*	+3121
**rs1063320**	*C*	*G*	+3142
**rs9380142**	*A*	*G*	+3187
**rs1610696**	*C*	*G*	+3196
**rs1233331**	*G*	*A*	+3227

^1^Castelli et al. (2017). *Del*: +2960 or 14-bp deletion and *Ins*: +2960 or 14-bp insertion.

The reverse sequence-specific oligonucleotide (rSSO) typing was used as the first step in *MICA* genotyping, according to the manufacturer’s recommendations, as outlined in the LABType MICA kit (One Lambda Inc., Canoga Park, CA, USA). This reaction was analyzed through a flow fluorimeter (LABScan 100, Austin, TX, USA), which employs LUMINEX technology. Analyses were performed with HLA Fusion Software, and *MICA* genotypes were discriminated according to *MICA-129 Val/Met* polymorphism as to the presence or absence (termed wild type in relation to *MICA A5*.*1* or *Wt* in this study) of *MICA A5*.*1* allelic variation at exon 5.

The *NKG2D* gene was genotyped using TaqMan allelic discrimination methodology and KLRC4-KLRK1 TaqMan SNP Genotyping Assays (product number: C_9345347_10, Applied Biosystems). The SNP-based genotyping was specific to detect a G>C (dbSNP: *rs1049174*) replacement and discriminate the allelic haplotype*s HNK1* and *LNK1*.

### ELISA for sHLA-G and sMICA

Soluble HLA-G concentrations were evaluated by a specific sandwich ELISA in plasma, using MEM-G/9 [38], anti-human β2-microglobulin as capture and detection antibodies, respectively [39]. This reaction recognizes sHLA-G1 and HLA-G5 isoforms. Similarly, sMICA was measured using a commercial kit according to the manufacturer’s protocol (DuoSet MICA ELISA, R & D Systems, Minneapolis, MN, USA). Plasma samples and calibrators were added to each well (100 μL) in duplicate to perform sMICA and sHLA-G measurements. The final concentration was determined from the optical density compared to standard curves (SD). The lower limit of detection for sHLA-G was 6.25 ng/mL and for sMICA was 31.25 pg/mL.

### Data analysis

Genotypes obtained for *HLA-G* (3’-UTR and coding sequence regions), *MICA* (*MICA-129 Val/Met* and *MICA A5*.*1/Wt*) and *NKG2D* (*HNK1* or *LNK1*) were analyzed for different genetic parameters.

The ELB algorithm was used to infer haplotypes for *HLA-G* and *MICA* for each individual, performing two runs, considering each gene individually. *HLA-G* 3’-UTR haplotypes were denominated according to the most recent UTR nomenclature, described by Castelli et al. [19]. The best phase (with reliability above 95%) obtained for *HLA-G* and *MICA* genes separately and individually for each individual was then used to perform analyses including *NKG2D* genotypes. The frequencies of each variation were computed as absolute and relative frequency. Also, the Hardy-Weinberg equilibrium was tested by the exact test of Guo and Thompson [40], for the control group.

Linkage disequilibrium (LD) parameters (*p* or LOD, D’ and r^2^) were also estimated and calculated for intragenic variations of *MICA* and *HLA-G* in each group (Ct, CKD, KTN and KTR patients) and considering all individuals. All parameters were estimated using ARLEQUIN 3.5 software [41], and LD was also analyzed using Haploview software [42].

Fisher’s Exact Test (FET) was performed to infer associations among the analyzed genes (alleles and defined haplotypes) and two pathological conditions (first, the risk for chronic kidney disease and second, the risk for kidney allograft rejection). Logistic regression was chosen to confirm predictions observed in FET. The case-control analysis was first performed without adjustment for specific variables (e.g. patients’ sex frequencies, donor’s age and frequencies of *HLA-B* mismatches); after that, adjustments were made to confirm the observed associations.

Furthermore, the amounts of sHLA-G and sMICA obtained for plasma samples from Ct (n = 75) were compared with CKD patients (n = 56). Pre-transplant plasma samples from KTR and KTN patients (n = 26) were also compared. These analyses included a Mann-Whitney U test and median evaluations. A Kruskal-Wallis test was performed to analyze differences of expression among allelic variations. All association analyses were performed using the software IBM SPSS version 25.

## Results

### *HLA-G* and *MICA* haplotypes definition for association analysis and description of new *HLA-G* haplotypes

**Fig 1:**
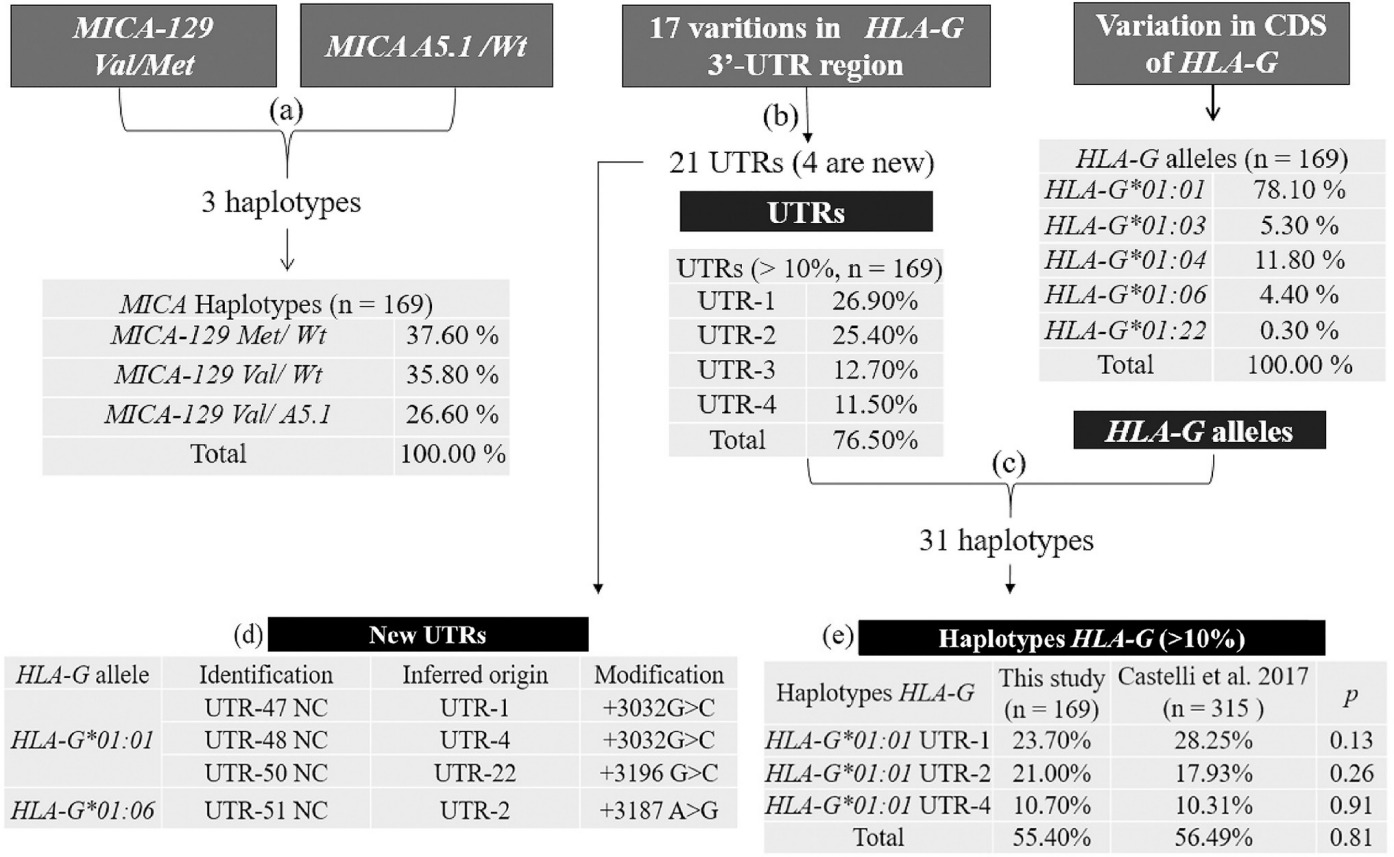
The three steps (a, b and c) of haplotype inference, *HLA-G* allele frequencies, and haplotype frequencies found in all samples (n = 169). Haplotype inferences: *MICA-129 Val/Met* and *MICA A5*.*1/Wt* **(a)**, between 17 variations found in 3’-UTR of the *HLA-G* gene **(b)**, and *HLA-G* UTRs and alleles **(c)**. New UTRs described and their inferred origin **(d)**. Similarity of frequencies of *HLA-G* haplotypes with published data from a population in São Paulo [18] **(e)**. *Wt*: wild type, which does not show *MICA A5*.*1* variation. CDS: coding DNA sequence (exon 2, 3 and 4 of the *HLA-G* gene). *Del*: +2960 or 14-bp deletion and *Ins*: +2960 or 14-bp insertion. NC: new composition.

Three haplotype inferences were performed between: (Fig 1a) *MICA-129 Val/Met* with *MICA A5*.*1/Wt*; (Fig 1b) the 17 variations found in 3’-UTR of the *HLA-G* gene, after identification according to UTR nomenclature [19]; and (Fig 1c) *HLA-G* UTRs with alleles.

Four new haplotypes for *HLA-G* 3’-UTR were described, termed UTR-47^NC^, UTR-48^NC^, UTR-50^NC^ and UTR-51^NC^ (Fig 1d). Also, the following five previously described variations [19] were found to be monomorphic, but with no SNPid (identity): +3044 (A), +3052 (C), +3092 (G), +3107 (C) and +3111 (G). The most frequent *HLA-G* haplotypes inferred for *HLA-G* alleles with UTRs were compared to published data and did not differ in their frequencies (Fig 1e).

These analyses were also done for *HLA-G* and *MICA*; however, 51.76% of the inferred haplotypes had less than 95% reliability and therefore they could not be included in other analyses. Seven individuals did not achieve reliability in haplotype UTR inference and were excluded.

The other inferences originated haplotypes with 95% reliability or higher. The haplotypes formed by inferences were 21 for *HLA-G* UTR, 31 for *HLA-G* coding DNA sequence plus UTR, and 3 for *MICA*. This data was used to perform case-control and LD analysis. Still, the polymorphic variations observed for the 3’-UTR of the *HLA-G* gene, the *HLA-G* alleles, *MICA* variations, and *NKG2D* haploblocks were analyzed for Hardy-Weinberg Equilibrium in the control group, and all loci were in equilibrium (see S1 Table).

### Association of HLA-G genotypes in CKD and KT patients

Case-control association analyses were performed through a contingency table to first detect significant relationships of alleles nucleotide variations, genotypes and/or previously defined haplotypes. All description of alleles and genotypes frequencies with their association results are respectively described in S2 and S3 Tables. Then, we applied the logistic regression analysis to endorse the primary association.

The *HLA-G* haplotype HLA-G*01:*01* UTR-4 (*p* = 0.035) (Table 3) and the *MICA-129 Val/Met* (*p* = 0.043, OR = 0.519, 95% CI = 0.280–0.962) genotype were found to be protective factors for the control group *versus* CKD patients, according to FET. After adjustments for age and sex, the described association with the *MICA-129 Val/Met* genotype was no longer significant (*p* = 0.131), the association with the *HLA-G* haplotype was maintained (*p* = 0.032, OR = 0.480, 95% CI = 0.199–0.961), although in the logistic regression analysis was found that neither association was significant.

**Table 3:** Summarized FET results.

**A**								
	**Ct—2n = 150**	**CKD—2n = 188**	**FET results**			**After adjustment for sex and age**		
Genetic factors	Relative frequency (%)		*p*	OR	95% CI	*p*	OR	95% CI
HLA-G*01:*01* UTR-4 haplotype	14.67	7.45	0.035	0.468	0.231–0.950	0.032	0.480	0.199–0.961
*MICA-129 Val/Met* genotype	53.33	37.23	0.043	0.519	0.280–0.962	0.131	---	---
**B**								
	**KTN—n = 36**	**KTR—n = 28**	**FET results**			**After adjustment for sex**		
Genetic factors	Relative frequency (%)		*p*	OR	95% CI	*p*	OR	95% CI
*+3187 AG*	25.00	53.57	0.037	3.461	1.201–9.978		---	---
*+3142 CG*	25.00	67.86	<0.001	6.333	2.120–18.924	0.013	5.357	1.417–20.261
*+3010 CG*	25.00	67.86	<0.001	6.333	2.120–18.924	0.013	5.357	1.417–20.261
*+3010 GG*	33.33	10.71	0.041	0.240	0.060–0.957	0.041	0.136	0.016–1.178

FET: Fisher’s Exact Test. Ct: Control group. CKD: Patients with chronic kidney disease. OR: *Odds Ratio*. CI: Confidence Interval.

For the KTR patients, the risk associations (Table 3) were found for genotypes *+3187 AG* (*p* = 0.037), *+3142 CG* (*p* < 0.001) and *+3010 CG* (*p* < 0.001). A protective association was found for *+3010 GG* (*p* = 0.041). After adjustments for the patients’ sex frequencies, donor’s age and *HLA-B* mismatches, the risk association of *+3010 CG* and *+3142 CG* was maintained (both with *p* = 0.013, OR = 5.357, 95% CI = 1.417–20.261), as well as a trend toward *+3010 GG* as a protective factor (*p* = 0.041, OR = 0.136, 95% CI = 0.016–1.178).

In the logistic regression (Table 4), including all loci, the genotype *+3010 CG* was confirmed as a risk factor and also *+3010 GG* as a protective factor for kidney allograft rejection (*p* = 0.004 for the categorical variable *HLA-G* +3010 included in predicted model). The model had a prediction power of 71.88% as is described in Table 5. This association was maintained even after adjustment (*p* = 0.038). Information concerning the observed and predicted frequencies for rejection by logistic regression can be seen in S4 Table. For the KTR patients was not detected association at the allelic level.

**Table 4:** Logistic regression analysis of kidney-transplant patients for rejection.

Predictor	β	SE β	Wald x2	*p*	e^β^ (*OR*)	95% CI for e^β^	
						Lower	Upper
*HLA-G* +3010 (Categorical variable)	---	---	11.054	0.004	---	---	---
*HLA-G* +3010 *CG*	1.664	0.630	6.969	0.008	5.278	1.535	18.148
*HLA-G* +3010 *GG*	-0.470	0.806	0.340	0.560	0.625	0.129	3.035
Constant	-0.916	0.483	3.598	0.058	0.400		
**Overall model evaluation**	**Null model**	**Predicted model**					
-2 Log likelihood	87.720	75.304					
Wald test	0.995	3.598					
Coefficient constant	0.251	-0.916					
**Goodness-of-fit test**	***p***						
Hosmer & Lemeshow	1.000						
Cox and Snell *R*	0.176						
Nagelkerke *R2*	0.236						

Binary Logistic Regression (method: forward stepwise conditional). Analysis performed with genotypes for KTN (coded as “0”; n = 36) *versus* KTR (coded as “1”; n = 28). Predicted logit (equation) of rejection = (-0.916) + (1.664)* (*HLA-G* +3010 *CG*) + (-0.470) * (*HLA-G* +3010 *GG*). The contrast of categorical variables and their references were respectively the indicator and the last subcategory according to codes described in available file “Data set.xlsx”. *HLA-G* +3010 categorical variable had as reference *HLA-G* +3010 *CC* genotype. KTN: Kidney-transplant patients with no rejection. KTR: Kidney-transplant patients who developed episodes of rejection. *OR*: *Odds Ratio*. CI: Confidence Interval.

**Table 5:** The observed and predicted frequencies for rejection by logistic regression with cutoff of 0.50.

	**Predicted**		
**Observed**	No	Yes	% Correct
**No**	27	9	75.00
**Yes**	9	19	67.86
**Overall % correct**			71.88

Sensitivity = 19/ (9+19) % = 67.86%. Specificity = 27/ (27+9) % = 75.00%. False positive = 9/ (9+19) % = 32.14%. False negative = 9/ (9+27) % = 25.00%.

### LD evaluation of *HLA-G* and *MICA* genes among case-control groups

LD analyzes were performed with the purpose of clarifying whether or not associations between polymorphisms would differ between the case-control groups (Ct and CKD, KTN and KTR). These associations could be determined by unique events arising from pathological states (CKD and KTR patients), contributing to an understanding of their origins. Therefore, this analysis was first performed separately for each group and compared. After the analysis was performed including all individuals.

For each group and for all sample, the estimated linkage disequilibrium did not diverge, nor did it diverge with respect to the variations in *cis*. All polymorphic sites found in 3’-UTR of the *HLA-G* gene showed a strong LD between them, except for the last polymorphism at position +3227.

Of all the analyses performed for each group (Ct, CKD, KTN and KTR), the highest LD was found in the *HLA-G* gene between positions +3010 and +3142, +3142 and 14-bp *Ins/Del*, and *MICA-129 Val/Met* and *MICA A5*.*1/Wt*, as summarized in Table 6 and extended in S5 Table.

**Table 6:** The most significant results for *HLA-G* 3’-UTR and *MICA* linkage disequilibrium for all groups (Ct, CKD, KTN and KTR).

Locus 1	Locus 2	D’	LOD	r^2^	Variants in *cis*	
**+3010 G>C**	+3142 C>G	1.000	7.90 to 39.27	0.87 to 1.000	*+3010 C* and *+3142 G*	*+3010 G* and *+3142 C*
***Ins/Del* 14bp**	+3142 C>G	0.91 to 1.00	5.06 to 18.86	0.42 to 0.62	14-bp *Ins* and *+3142 G*	14-bp *Del* and *+3142 C*
***MICA-129 Val/Met***	*MICA A5*.*1/Wt*	1.000	2.22 to 6.71	0.19 to 0.27	*MICA-129 Met* and *MICA Wt*	*MICA-129 Val* and *MICA A5*.*1*

The significant linkage disequilibrium had an LOD > 3.000 and *p* < 0.05. LOD: is the log of the likelihood odds ratio. Ct: Control group. CKD: Patients with chronic kidney disease. KTN: Kidney-transplant patients with no rejection. KTR: Kidney-transplant patients who developed episodes of rejection. *Wt*: wild type, which does not show *MICA A5*.*1* variation. *Del*: +2960 or 14-bp deletion and *Ins*: +2960 or 14-bp insertion.

The LDs inferred for *HLA-G* alleles and UTRs were also strong, especially among the HLA-G*01:*03* with UTR-5 or UTR-13, and the HLA-G*01:*04* with UTR-3 (for all LD results see S5 Table). These haplotypes are also found in other populations [19].

### Soluble HLA-G and MICA phenotypes association

**Fig 2:**
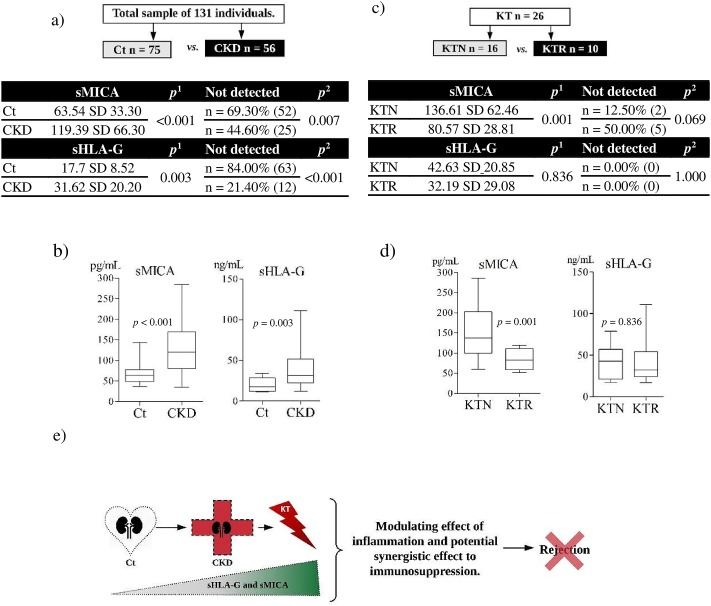
Case-control analysis of sMICA and sHLA-G production. Analyses of sMICA and sHLA-G were performed for control (Ct) *versus* chronic kidney disease (CKD) **(a-b)**, and after kidney transplant (KT) in patients with (KTR) and without (KTN) episodes of rejection **(c-d)**. The production of regulatory molecules, sHLA-G and sMICA, is stimulated in a pathological condition, such as in patients with chronic kidney disease (CKD), but generally not in individuals in homeostasis, as observed in Ct. Once the CKD is established, the regulatory molecules in the pre-transplant can act in the post-transplant period as enhancers of immunoregulation, leading to allograft acceptance **(e)**. Mann-Whitney test^1^. Chi-square test^2^. SD: Standard deviation.

The quantitative analyses of sHLA-G and sMICA were performed to evaluate differences between two conditions, comparing patients with chronic kidney disease (CKD) with controls (Ct) and patients who had undergone a kidney transplant and developed rejection (KTR) with those who did not (KTN) (Fig 2a and 2c).

The first evaluation showed significantly higher levels of both molecules in the CKD group compared to the Ct group (sMICA median for CKD: 119.39 pg/mL and Ct: 63.54 ng/mL, *p* <0.001; sHLA-G median for CKD: 31.62 pg/mL and Ct: 17.70 ng/mL, *p* = 0.003). Also, the percentage of individuals for which neither molecule had no detection (under the limit of detection of 6.25 ng/mL for sHLA-G and 31.25 pg/mL for sMICA) was higher for the Ct compared to the CKD (Fig 2a and 2b).

Comparison between the KTN and KTR groups showed a difference for sMICA (median for KTN: 136.61 pg/mL and for KTR: 80.57 pg/mL, *p* = 0.001) but no difference for sHLA-G (median for KTN: 42.63 ng/mL and for KTR: 32.19 ng/mL, *p* = 0.836). The percentage of individuals with no detectable sMICA was higher in the KTR group (Fig 2c and 2d).

*MICA* haplotypes were analyzed in association with sMICA quantification, and no differences were detected (*p* = 0.327). sHLA-G and genetic variations were evaluated for the most frequently detected alleles (HLA-G*01:*01*, HLA-G*01:*04* and HLA-G*01:*03*) and UTRs (UTR-1, -2, -3, -4 and -5), but no differences were observed (respectively, *p* = 0.448 and *p* = 0.585, more information see S7 Table and S1 Fig).

## Discussion

Case-control analysis included *HLA-G*, *MICA* and *NKG2D* genotype data and quantification of soluble molecules (sHLA-G and sMICA) involved in immunomodulation of inflammatory response. Regarding the association tests performed for chronic kidney disease patients, the HLA-G*01:*01/*UTR-4 was found to be a protective factor for the development of chronic kidney disease (FET analysis). In particular, UTR-4 has previously been related to higher stability of *HLA-G* transcripts and immunoregulation in successful pregnancies [43]. This suggest the HLA-G*01:*01/*UTR-4 could be a protection against inflammatory conditions and pathological states, not just found in women with secondary recurrent miscarriage, but also in CKD patients.

For the kidney-transplant patients, the *+3010 CG* genotype was associated with kidney allograft rejection. This SNP has the strongest LD with the +3142 position. Also, the 14-bp *Ins/Del* had high LD with +3142 and +3010 SNPs. The variations found in *cis* were related to their effect when inherited together, as previously described for HLA-G [44–49]. Thus, the composition possibly associated with the risk of kidney allograft rejection by the decrease of HLA-G expression would be *+3010 C*, *+3142 G* and 14-bp *Ins*, whereas *+3010 G* with *+3142 C*, and *+3142 C* with 14-bp *Del* would be associated with an increase.

The presence of *+3010 C* would act as a negative factor for the allograft outcome (even in the heterozygote), as it is linked to the *+3142 G* variation that promotes greater affinity of *HLA-G* transcripts to microRNAs, leading to a decreased HLA-G expression [50]. This effect would be more evident in the presence of 14-bp *Ins*, from which unstable transcripts originate [44]. The analyses were not able to detect a direct relation regarding the *+3010 CC* and rejection, but twice as many patients who developed episodes of rejection have this genotype compared to the frequencies of *+3010 GG* patients, which was associated as a protection factor. The association of +3142 with 14-bp *Ins/Del* has been the subject of many studies aiming to identify risk factors [45,46,49,51]. However, we suggest that +3010 is a marker to predict the simultaneous presence of *+3142 G* with 14-bp *Ins*.

Concerning the sMICA and sHLA-G production, those molecules functionally preserve the expression of a similar inflammatory condition [11,52]. Our analysis showed that both molecules are more highly expressed in patients in a stressful condition, such as chronic kidney disease (CKD), compared to the control. Also as expected, in the Ct group the non-detection of sMICA and sHLA-G was more common than in the CKD group, which reflects the stimulation of immunological changes and production of regulatory molecules (sHLA-G and sMICA) under a pathological condition.

In relation to the kidney-transplant patients and soluble molecules, there was evidence of higher production of sMICA in the KTN compared to the KTR group, who developed rejection. Therefore, once the chronic disease is established in the patient, the production of regulatory soluble molecules in the pre-transplant period acts as an immunoregulatory effect contributing to the acceptance of the allograft (Fig 2e). Thus, the pre-transplant status of sMICA can potentially influence the successful outcome of the kidney transplantation. Particularly, the evaluation of MICA expression need to take into account its isoform (membrane bound or soluble) related to its function (activation or inhibition of NK cells), thus MICA mRNA levels [5] may be a confounding manner to evaluate this biomarker.

Regarding to the difference in the sMICA and sHLA-G production, no allelic differentiation was observed. This demonstrates that the joint analysis of other forms, such as those bound to the membrane, is necessary to perform this type of analysis.

Finally, considering the Brazilian population is one of the most admixed in the world [53], new genetic variations can be found throughout the country, as well as similarities. In this study, the *MICA*, *NKG2D* and *HLA-G* genes evaluation also comprised part of this diversity. For the *HLA-G* gene, a total of 21 UTRs were observed, four of them new. The *HLA-G* haplotype frequencies did not differ from data reported by Castelli et al. (2014) [54]. For *MICA*, 3 haplotypes were detected and the *MICA-129 Met* with *MICA A5*.*1* was not present. Of the *HLA-G* haplotypes, the combinations of HLA-G*01:*03*/UTR-5 or /UTR-13 with HLA-G*01:*04*/UTR-3 were most frequent, which is in accordance with the main complete extended haplotypes previously described [19].

## Conclusion

The increased production of immunoregulatory molecules, such as sMICA and sHLA-G, was associated with chronic kidney disease. Still, a higher sMICA production in the pre-transplant period was found to be associated with a better kidney allograft outcome.

Association of genetic factors with different quantifications of sMICA and sHLA-G already reported in other works [14,17,18,27–30], were not observed here. However, the +3010 SNP (rs1710) in *HLA-G* gene, showed potential as a molecular marker for the characterization of +3142 SNP and 14-bp *Ins/Del* variation, all related to changes in HLA-G production and to kidney allograft rejection. Thus, this finding indicate that phenotype and genotype association analysis should include quantification of immunoregulatory molecules in the cell membrane-bound and soluble isoforms.

Moreover, our study described new *HLA-G* UTR haplotypes, which reinforces the importance of conducting further studies that aim to better characterize the diversity of the Brazilian population.

## Supporting information

S1 Table Analyses of Hardy-Weinberg equilibrium performed for HLA-G alleles and polymorphisms in 3’-UTR region, MICA and NKG2D variations in the control group (n = 75). Positions +3001, +3032, +3044, +3052, +3092, 3107, +3111 and +3121 are monomorphic sites in the control group. 1HLA-G haplotypes: inferred composition through the ELB algorithm of HLA-G alleles with determined UTRs. Wt: wild type, which does not show MICA A5.1 variation. SD: Standard deviation.
(PDF)

S2 Table Observed allele frequencies and Fischer’s Exact Test results for the *HLA-G*, *MICA* and *NKG2D* genes. Ct: Control group. CKD: Patients with chronic kidney disease. KTN: Kidney-transplant patients with no rejection. KTR: Kidney-transplant patients who developed episodes of rejection. Wt: wild type, which does not show *MICA A5*.*1* variation. *Del*: +2960 or 14-bp deletion and *Ins*: +2960 or 14-bp insertion. ^1^ Zero or not sufficient frequency to perform the calculation.
(PDF)

S3 Table Observed genotype frequencies and Fischer’s Exact Test results for *HLA-G*, *MICA* and *NKG2D* genes. Ct: Control group. CKD: Patients with chronic kidney disease. KTN: Kidney-transplant patients with no rejection. KTR: Kidney-transplant patients who developed episodes of rejection. *Wt*: wild type, which does not show *MICA A5*.*1* variation. Del: +2960 or 14-bp deletion and *Ins*: +2960 or 14-bp insertion.
(PDF)

S4 Table Predicted probability of rejection for kidney transplant patients. (PDF)

S5 Table The linkage disequilibrium results for HLA-G 3'-UTR and MICA in each group (Ct, CKD, KTN and KTR). Analysis performed in Haploview. The linkage disequilibrium (LD) considered significant had an LOD > 3.000. LOD: is the log of the likelihood odds ratio. 95% CI relative to D’: confidence interval. Ct: Control group. CKD: Patients with chronic kidney disease. KTN: Kidney-transplant patients with no rejection. KTR: Kidney-transplant patients who developed episodes of rejection. Wt: wild type, which does not show MICA A5.1 variation. Del: +2960 or 14-bp deletion and Ins: +2960 or 14-bp insertion.
(PDF)

S6 Table The linkage disequilibrium results for HLA-G 3'-UTR and MICA in each group (Ct, CKD, KTN and KTR). The inferences of HLA-G haplotype through ELB algorithm of HLA-G alleles with determined UTRs. Analysis performed in Arlequin. The significant linkage disequilibrium had a p < 0.05. Ct: Control group. CKD: Patients with chronic kidney disease. KTN: Kidney-transplant patients with no rejection. KTR: Kidney-transplant patients who developed episodes of rejection.
(PDF)

S7 Table Association analysis performed between soluble molecules and genetic factors. Q1: First Quartile. Q3: Third Quartile.
(PDF)

S8 Table Data set.xlsx. (a) Data_set_CKD_Ct_n_169. (b) Code_book_Data_set_CKD_Ct. (c) Data_set_KTR_KTN_n_64. (d) Code_book_Data_set_KTR_KTN.
(XLSX)

S1 Fig Representation of median, first and third quartile from soluble molecules associated with genetic factors. Soluble MICA (sMICA) and *MICA* haplotypes (MICA-129 Val/Met and MICA A5.1/Wt) association (*p* = 0.327) **(a)**. Soluble HLA-G and *HLA-G* alleles association (*p* = 0.448) **(b)**. Soluble HLA-G and most frequent *HLA-G* UTRs association (*p* = 0.585) **(c)**.
(TIF)
