# Supplementary material for: The association of HLA-G polymorphisms and the synergistic effect of sMICA and sHLA-G with chronic kidney disease and allograft acceptance
Source: PLoS One. 2019 Feb 22;14(2):e0212750. doi: 10.1371/journal.pone.0212750 (PMC6386361; doi:10.1371/journal.pone.0212750)
Supplement: S1 Table — Positions +3001, +3032, +3044, +3052, +3092, 3107, +3111 and +3121 are monomorphic sites in the control group. 1HLA-G haplotypes: inferred composition through the ELB algorithm of HLA-G alleles with determined UTRs. Wt: wild type, which does not show MICA A5.1 variation. SD: Standard deviation. (PDF) [file pone.0212750.s001.pdf]

S1 Table. Analyses of Hardy-Weinberg equilibrium performed for *HLA-G* alleles and polymorphisms in 3'-UTR region, *MICA* and *NKG2D* variations in the control group (n = 75).

Locus	Observed heterozygosis	Expected heterozygosis	p	*SD*
HLA-G 3'-UTR polymorphisms				
HLA-G +2960	0.547	0.486	0.341	<0.001
HLA-G +3003	0.280	0.261	1.000	<0.001
HLA-G +3010	0.507	0.503	1.000	<0.001
HLA-G +3027	0.120	0.114	1.000	<0.001
HLA-G +3035	0.147	0.181	0.144	<0.001
HLA-G +3142	0.520	0.502	0.817	<0.001
HLA-G +3187	0.373	0.367	1.000	<0.001
HLA-G +3196	0.493	0.433	0.285	<0.001
HLA-G +3227	0.133	0.125	1.000	<0.001
HLA-G UTRs	0.867	0.821	0.387	<0.001
HLA-G alleles	0.333	0.356	0.246	<0.001
HLA-G haplotypes[1]	0.907	0.85	0.663	<0.001
MICA-129 Val/Met	0.653	0.661	0.309	<0.001
MICA A5.1/Wt	0.333	0.400	0.156	<0.001
MICA haplotypes	0.533	0.488	0.480	<0.001
NKG2D	0.507	0.456	0.443	<0.001

Positions +3001, +3032, +3044, +3052, +3092, 3107, +3111 and +3121 are monomorphic sites in the control group. [1]*HLA-G* haplotypes: inferred composition through the ELB algorithm of *HLA-G* alleles with determined UTRs. *Wt*: wild type, which does not show *MICA A5.1* variation. SD: Standard deviation.
