# Supplementary material for: The association of HLA-G polymorphisms and the synergistic effect of sMICA and sHLA-G with chronic kidney disease and allograft acceptance
Source: PLoS One. 2019 Feb 22;14(2):e0212750. doi: 10.1371/journal.pone.0212750 (PMC6386361; doi:10.1371/journal.pone.0212750)
Supplement: S2 Table — Ct: Control group. CKD: Patients with chronic kidney disease. KTN: Kidney-transplant patients with no rejection. KTR: Kidney-transplant patients who developed episodes of rejection. Wt: wild type, which does not show MICA A5.1 variation. Del: +2960 or 14-bp deletion and Ins: +2960 or 14-bp insertion. 1 Zero or not sufficient frequency to perform the calculation. (PDF) [file pone.0212750.s002.pdf]

S2 Table. Observed allele frequencies and Fischer's Exact Test results for the *HLA-G*, *MICA* and *NKG2D* genes.

Position	Variation	Ct - 2n = 150	CKD - 2n = 188		KTN - 2n = 72	KTR - 2n = 56	
***HLA-G* 3'-UTR**		**Relative frequency (%)**		***p***	**Relative frequency (%)**		***p***
+2960	14-bp *Del*	59.30	62.80	0.574	62.50	62.50	1.000
	14-bp *Ins*	40.70	37.20	0.520	37.50	37.50	1.000
+3001	*C*	100.00	99.50	0.371	---[1]	---[1]	---
	T	0.00	0.50	1.000	---[1]	---[1]	---
+3003	*C*	15.30	10.10	0.148	11.10	8.80	0.774
	T	84.70	90.90	0.184	88.90	91.20	0.685
+3010	*G*	49.30	46.30	0.576	45.80	55.60	0.373
	C	50.70	53.70	0.586	54.20	44.40	0.893
+3027	*C*	94.00	95.70	0.618	94.40	96.40	1.000
	A	6.00	4.30	0.466	5.60	5.60	1.000
+3032	*G*	100.00	98.90	0.205	---[1]	---[1]	---
	C	0.00	1.10	1.000	---[1]	---[1]	---
+3035	*C*	90.00	87.80	0.518	87.50	91.10	0.521
	T	10.00	12.20	0.604	12.50	8.90	0.580
+3142	*C*	47.30	47.30	0.999	45.80	48.20	0.789
	G	52.70	52.70	0.999	54.20	51.80	0.859
+3187	*A*	76.00	67.60	0.088	70.80	66.10	0.701
	G	24.00	32.40	0.092	29.20	33.90	0.564
+3196	*C*	68.70	72.3	0.473	69.40	69.60	0.981
	G	31.30	27.70	0.461	30.60	30.40	1.000
+3227	*G*	93.30	97.30	0.109	95.80	98.20	0.443
	A	6.70	2.70	0.075	4.20	1.80	0.631
***HLA-G* UTR**							
	UTR-1	24.00	29.26	0.324	29.17	25.00	1.000
	UTR-2	28.67	22.87	0.168	23.61	23.21	1.000
	UTR-3	10.67	14.36	0.396	15.28	12.50	0.799
	UTR-4	15.33	8.51	0.060	8.33	8.93	1.000
	UTR-18	2.10	6.70	0.053	---[1]	---[1]	---
***HLA-G* Alleles**							
	*HLA-G*01:01*	79.33	77.13	0.692	0.79	0.79	1.000
	*HLA-G*01:03*	4.00	6.38	0.466	0.07	0.02	0.230
	*HLA-G*01:04*	10.67	12.77	0.613	0.08	0.02	0.268
	*HLA-G*01:06*	6.00	3.19	0.288	0.04	0.04	0.268
	*HLA-G*01:22*	0.00	0.53	1.000	0.01	0.00	1.000
***HLA-G* Haplotypes**							
	HLA-G*01:01 UTR-1	22.67	24.47	0.702	25.00	23.21	0.839
	HLA-G*01:01 UTR-2	21.33	20.74	1.000	19.44	25.00	0.520
	HLA-G*01:01 UTR-3	4.00	6.38	0.466	4.17	7.14	0.698
	HLA-G*01:01 UTR-4	14.67	7.45	0.035	9.72	7.14	0.755
	HLA-G*01:04 UTR-3	5.33	5.85	1.000	6.94	5.36	1.000
	*HLA-G*01:04* UTR-1	---[1]	---[1]	---	0.00	7.10	0.002
MICA							
	MICA-129 Val	58.70	65.40	0.215	65.3	53.60	0.205
	MICA-129 Met	41.30	34.60	0.202	34.70	46.40	0.180
	MICA A5.1	27.30	26.00	0.793	70.80	82.10	0.138
	MICA Wt	72.70	74.00	0.805	29.20	17.9	0.152
***MICA* Haplotype**							
	MICA-129 Met/ Wt	41.33	34.57	0.215	34.70	46.40	0.280
	MICA-129 Val/ Wt	31.33	39.36	0.139	36.10	35.70	1.000
	MICA-129 Val/ A5.1	27.33	26.06	0.805	29.20	17.90	0.152
NKG2D							
	LNK1	65.33	65.33	0.571	58.33	60.71	0.857
	HNK1	34.67	34.67	0.571	41.67	39.29	0.857

Ct: Control group. CKD: Patients with chronic kidney disease. KTN: Kidney-transplant patients with no rejection. KTR: Kidney-transplant patients who developed episodes of rejection. *Wt*: wild type, which does not show *MICA A5.1* variation. *Del*: +2960 or 14-bp deletion and *Ins*: +2960 or 14-bp insertion. [1] Zero or not sufficient frequency to perform the calculation.
