# Supplementary material for: The association of HLA-G polymorphisms and the synergistic effect of sMICA and sHLA-G with chronic kidney disease and allograft acceptance
Source: PLoS One. 2019 Feb 22;14(2):e0212750. doi: 10.1371/journal.pone.0212750 (PMC6386361; doi:10.1371/journal.pone.0212750)
Supplement: S3 Table — Ct: Control group. CKD: Patients with chronic kidney disease. KTN: Kidney-transplant patients with no rejection. KTR: Kidney-transplant patients who developed episodes of rejection. Wt: wild type, which does not show MICA A5.1 variation. Del: +2960 or 14-bp deletion and Ins: +2960 or 14-bp insertion. (PDF) [file pone.0212750.s003.pdf]

S3 Table. Observed genotype frequencies and Fischer's Exact Test results for *HLA-G*, *MICA* and *NKG2D* genes.

Position	Genotype	Ct - n = 75	CKD - n = 94		KTN - n = 36	KTR - n = 28	
HLA-G genotypes with 3'-UTR		Relative frequency (%)		*p*	Relative frequency (%)		*p*
+2960	14-bp *Del* / 14-bp *Del*	32.00	40.43	0.267	41.67	39.29	1.000
	14-bp *Del* / 14-bp *Ins*	54.67	44.68	0.218	41.67	46.43	0.801
	14-bp *Ins* / 14-bp *Ins*	13.30	14.89	0.827	16.67	14.29	1.000
+3001	*C/ C*	100.00	98.94	1.000	100.00	100.00	---
	C/ T	0.00	1.06	1.000	0.00	0.00	---
	T/ T	0.00	0.00	---	0.00	0.00	---
+3003	*C/C*	1.33	1.06	1.000	2.78	0.00	1.000
	C/T	28.00	18.09	0.141	16.67	17.86	1.000
	T/ T	70.68	80.85	0.146	80.56	82.14	1.000
+3010	*G/ G*	24.00	23.40	1.000	33.33	10.71	0.041
	G/ C	50.67	45.74	0.539	25.00	67.86	<0.001
	C/ C	25.33	30.90	0.494	41.67	21.43	0.111
+3027	*C/ C*	88.00	91.49	0.608	88.89	92.86	0.688
	C/ A	12.00	8.51	0.608	11.11	7.14	0.688
	A/ A	0.00	0.00	---	0.00	0.00	---
+3032	*G/ G*	100.00	98.94	1.000	100.00	100.00	---
	G/ C	0.00	0.00	---	0.00	0.00	---
	C/ C	0.00	1.06	1.000	0.00	0.00	---
+3035	*C/ C*	82.67	79.79	0.696	80.56	85.71	0.743
	C/ T	14.67	15.96	0.834	13.89	10.71	1.000
	T/ T	2.67	4.26	0.694	5.56	3.57	1.000
+3142	*G/ G*	26.67	29.79	0.732	41.67	17.86	0.058
	G/ C	52.00	45.74	0.442	25.00	67.86	<0.001
	C/ C	21.33	24.47	0.714	33.33	14.29	0.144
+3187	*G/ G*	5.33	13.83	0.077	16.67	7.14	0.448
	G/ A	37.33	37.23	1.000	25.00	53.57	0.036
	A/ A	57.33	48.94	0.283	58.33	39.29	0.207
+3196	*G/ G*	6.67	9.57	0.582	11.11	10.71	1.000
	G/ C	49.33	36.17	0.117	38.89	39.29	1.000
	C/ C	44.00	54.26	0.216	50.00	50.00	1.000
+3227	*G/ G*	86.67	94.68	0.101	91.67	96.43	0.625
	G/ A	13.33	5.32	0.101	8.33	3.57	0.625
	A/ A	0.00	0.00	---	0.00	0.00	---
HLA-G genotypes with UTRs							
	UTR-1/ UTR-1	5.33	11.70	0.180	16.67	0.00	0.031
	UTR-1/ UTR-2	13.33	11.70	0.817	5.56	21.43	0.124
	UTR-1/ UTR-3	6.67	9.57	0.582	2.78	17.86	0.078
	UTR-1/ UTR-4	9.33	6.38	0.566	11.11	3.57	0.375
	UTR-1/ UTR-5	0.00	3.19	0.255	0.00	0.00	---
	UTR-1/ UTR-6	1.33	2.13	1.000	2.78	3.57	1.000
	UTR-1/ UTR-7	1.33	1.06	1.000	0.00	3.57	0.437
	UTR-1/ UTR-8	2.67	0.00	0.195	0.00	0.00	---
	UTR-1/ UTR-10	0.00	1.06	1.000	2.78	0.00	1.000
	UTR-1/ UTR-16	0.00	1.06	1.000	0.00	3.57	0.437
	UTR-1/ UTR-18	2.67	0.00	0.195	0.00	0.00	---
	UTR-1/ UTR-51	0.00	1.06	1.000	2.78	0.00	1.000
	UTR-2/ UTR-2	5.33	6.38	1.000	2.78	10.71	0.310
	UTR-2/ UTR-3	9.33	8.51	1.000	8.33	7.14	1.000
	UTR-2/ UTR-4	10.67	5.32	0.249	5.56	7.14	1.000
	UTR-2/ UTR-5	2.67	1.06	0.585	0.00	0.00	---
	UTR-2/ UTR-6	1.33	1.06	1.000	0.00	3.57	0.437
	UTR-2/ UTR-7	1.33	3.19	0.630	5.56	3.57	1.000
	UTR-2/ UTR-10	1.33	2.13	1.000	5.56	0.00	0.500
	UTR-2/ UTR-18	6.67	3.19	0.469	5.56	0.00	0.500
	UTR-2/ UTR-50	0.00	1.06	1.000	2.78	0.00	1.000
	UTR-3/ UTR-3	1.33	1.06	1.000	2.78	0.00	1.000
	UTR-3/ UTR-4	2.67	3.19	1.000	0.00	3.57	0.437
	UTR-3/ UTR-5	0.00	1.06	1.000	2.78	0.00	1.000
	UTR-3/ UTR-7	0.00	1.06	1.000	0.00	0.00	---
	UTR-3/ UTR-18	0.00	1.06	1.000	0.00	3.57	0.437
	UTR-4/ UTR-4	1.33	1.06	1.000	2.78	0.00	1.000
	UTR-4/ UTR-5	0.00	2.13	0.503	0.00	3.57	0.437
	UTR-4/ UTR-7	4.00	0.00	0.085	0.00	0.00	---
	UTR-4/ UTR-18	1.33	0.00	0.444	0.00	0.00	---
	UTR-5/ UTR-7	1.33	3.19	0.630	2.78	3.57	1.000
	UTR-5/ UTR-8	1.33	0.00	0.444	0.00	0.00	---
	UTR-5/ UTR-13	1.33	1.06	1.000	2.78	0.00	1.000
	UTR-5/ UTR-18	0.00	1.06	1.000	2.78	0.00	1.000
	UTR-6/ UTR-17	0.00	1.06	1.000	0.00	0.00	---
	UTR-6/ UTR-18	0.00	1.06	1.000	2.78	0.00	1.000
	UTR-7/ UTR-15	1.33	0.00	0.444	0.00	0.00	---
	UTR-7/ UTR-18	2.67	0.00	0.195	0.00	0.00	---
	UTR-47/ UTR-48	0.00	1.06	0.444	0.00	0.00	---

Ct: Control group. CKD: Patients with chronic kidney disease. KTN: Kidney-transplant patients with no rejection. KTR: Kidney-transplant patients who developed episodes of rejection. *Wt*: wild type, which does not show *MICA A5.1* variation. *Del*: +2960 or 14-bp deletion and *Ins*: +2960 or 14-bp insertion.

S3 Table. Observed genotype frequencies and Fischer's Exact Test results for *HLA-G*, *MICA* and *NKG2D* genes (continuation).

Position	Genotype	Ct - n = 75	CKD - n = 94		KTN - n = 36	KTR - n = 28	
HLA-G genotypes with alleles							
	*HLA-G*01:01/ HLA-G*01:01*	64.00	60.64	0.750	66.67	64.29	1.000
	*HLA-G*01:01/ HLA-G*01:03*	5.33	9.57	0.390	5.56	3.57	1.000
	*HLA-G*01:01/ HLA-G*01:04*	16.00	17.02	1.000	8.33	17.86	0.448
	*HLA-G*01:01/ HLA-G*01:06*	9.33	5.32	0.374	8.33	7.14	1.000
	*HLA-G*01:01/ HLA-G*01:22*	0.00	1.06	1.000	2.78	0.00	1.000
	*HLA-G*01:03/ HLA-G*01:03*	1.33	1.06	1.000	2.78	0.00	1.000
	*HLA-G*01:03/ HLA-G*01:04*	0.00	1.06	1.000	2.78	0.00	1.000
	*HLA-G*01:04/ HLA-G*01:04*	1.33	3.19	0.630	2.78	7.14	0.577
	*HLA-G*01:04/HLA-G*01:06*	2.67	1.06	0.585	0.00	0.00	---
MICA-129 Val/ Met genotypes							
	MICA-129 Val/ Val	32.00	46.81	0.059	41.67	32.14	0.603
	MICA-129 Val/ Met	53.33	37.23	0.043	47.22	42.86	0.803
	MICA-129 Met/ Met	14.67	15.96	0.834	11.11	25.00	0.188
MICA A5.1/Wt genotypes							
	MICA A5.1/ A5.1	10.67	8.51	0.792	5.56	3.57	1.000
	MICA A5.1/ Wt	33.33	35.11	0.871	47.22	28.57	0.197
	MICA Wt/ Wt	56.00	56.38	1	47.22	67.86	0.208
MICA genotypes with haplotypes							
	MICA Wt/Wt and *MICA-129 Met/ Met*	14.67	15.96	0.834	11.11	25.00	0.188
	MICA Wt/A5.1 and *MICA-129 Met/Val*	21.33	14.89	0.314	22.22	10.71	0.322
	MICA Wt/A5.1 and *MICA-129 Val/Val*	12.00	20.21	0.211	25.00	17.86	0.555
	MICA Wt/ Wt and *MICA-129 Val/ Val*	9.33	18.09	0.124	11.11	10.71	1.000
	MICA A51/ A5.1 and *MICA-129 Val/ Val*	10.67	8.51	0.792	5.56	3.57	1.000
	MICA Wt/Wt and *MICA-129 Met/ Val*	32.00	22.34	0.166	25.00	32.14	0.583
NKG2D genotypes							
	LNK1/ LNK1	40.00	40.43	1.000	33.33	39.29	0.793
	LNK1/ HNK1	50.67	43.62	0.438	50.00	42.86	0.620
	HNK1/ HNK1	9.33	15.96	0.253	16.67	17.86	1.000

Ct: Control group. CKD: Patients with chronic kidney disease. KTN: Kidney-transplant patients with no rejection. KTR: Kidney-transplant patients who developed episodes of rejection. *Wt*: wild type, which does not show *MICA A5.1* variation. *Del*: +2960 or 14-bp deletion and *Ins*: +2960 or 14-bp insertion.
