# Supplementary material for: The association of HLA-G polymorphisms and the synergistic effect of sMICA and sHLA-G with chronic kidney disease and allograft acceptance
Source: PLoS One. 2019 Feb 22;14(2):e0212750. doi: 10.1371/journal.pone.0212750 (PMC6386361; doi:10.1371/journal.pone.0212750)
Supplement: S4 Table — (PDF) [file pone.0212750.s004.pdf]

S4 Table. Predicted probability of rejection for kidney transplant patients.

Case number	*HLA-G* +3010 *CG* score β = 1.664	*HLA-G* +3010 GG score β = -0.470	Intercept = -0.916	Predicted probability of rejection for kidney transplant patients	Actual outcome (1 = Yes, 0 = No)
TXR01	0	0	-0.916	-0.916	0
TXR02	*GC*	0	-0.916	0.084	1
TXR03	*GC*	0	-0.916	0.084	1
TXR04	0	*GG*	-0.916	0.084	1
TXR05	0	0	-0.916	-0.916	0
TXR06	*GC*	0	-0.916	0.084	1
TXR07	*GC*	0	-0.916	0.084	1
TXR08	*GC*	0	-0.916	0.084	0
TXR09	*GC*	0	-0.916	0.084	1
TXR11	0	*GG*	-0.916	0.084	0
TXR13	0	*GG*	-0.916	0.084	0
TXR14	0	0	-0.916	-0.916	0
TXR15	0	0	-0.916	-0.916	0
TXR16	0	0	-0.916	-0.916	1
TXR17	0	*GG*	-0.916	0.084	0
TXR18	0	*GG*	-0.916	0.084	0
TXR19	0	*GG*	-0.916	0.084	0
TXR20	0	0	-0.916	-0.916	1
TXR21	0	0	-0.916	-0.916	0
TXR22	*GC*	0	-0.916	0.084	0
TXR23	0	0	-0.916	-0.916	0
TXR24	*GC*	0	-0.916	0.084	1
TXR25	0	0	-0.916	-0.916	0
TXR26	*GC*	0	-0.916	0.084	1
TXR27	*GC*	0	-0.916	0.084	0
TXR28	0	0	-0.916	-0.916	0
TXR29	0	*GG*	-0.916	0.084	0
TXR30	0	0	-0.916	-0.916	0
TXR31	*GC*	0	-0.916	0.084	0
TXR32	*GC*	0	-0.916	0.084	0
TXR33	*GC*	0	-0.916	0.084	1
TXR34	0	0	-0.916	-0.916	1
TXR35	*GC*	0	-0.916	0.084	1
TXR36	*GC*	0	-0.916	0.084	1
TXR37	*GC*	0	-0.916	0.084	1
TXR38	0	*GG*	-0.916	0.084	0
TXR39	*GC*	0	-0.916	0.084	0
TXR40	*GC*	0	-0.916	0.084	1
TXR41	*GC*	0	-0.916	0.084	1
TXR42	*GC*	0	-0.916	0.084	1
TXR43	0	0	-0.916	-0.916	0
TXR44	0	0	-0.916	-0.916	0
TXR45	0	*GG*	-0.916	0.084	1
TXR47	0	0	-0.916	-0.916	0
TXR48	0	0	-0.916	-0.916	0
TXR49	*GC*	0	-0.916	0.084	0
TXR50	0	*GG*	-0.916	0.084	0
TXR51	0	*GG*	-0.916	0.084	0
TXR52	*GC*	0	-0.916	0.084	0
TXR53	0	0	-0.916	-0.916	1
TXR54	0	0	-0.916	-0.916	0
TXR55	0	0	-0.916	-0.916	1
TXR56	*GC*	0	-0.916	0.084	1
TXR57	*GC*	0	-0.916	0.084	1
TXR58	0	0	-0.916	-0.916	1
TXR59	0	*GG*	-0.916	0.084	1
TXR60	*GC*	0	-0.916	0.084	1
TXR61	*GC*	0	-0.916	0.084	1
TXR62	*GC*	0	-0.916	0.084	1
TXR63	0	*GG*	-0.916	0.084	0
TXR64	*GC*	0	-0.916	0.084	0
TXR65	0	*GG*	-0.916	0.084	0
TXR66	0	0	-0.916	-0.916	0
TXR67	0	*GG*	-0.916	0.084	0
