# Supplementary material for: The association of HLA-G polymorphisms and the synergistic effect of sMICA and sHLA-G with chronic kidney disease and allograft acceptance
Source: PLoS One. 2019 Feb 22;14(2):e0212750. doi: 10.1371/journal.pone.0212750 (PMC6386361; doi:10.1371/journal.pone.0212750)
Supplement: S5 Table — Analysis performed in Haploview. The linkage disequilibrium (LD) considered significant had an LOD > 3.000. LOD: is the log of the likelihood odds ratio. 95% CI relative to D’: confidence interval. Ct: Control group. CKD: Patients with chronic kidney disease. KTN: Kidney-transplant patients with no rejection. KTR: Kidney-transplant patients who developed episodes of rejection. Wt: wild type, which does not show MICA A5.1 variation. Del: +2960 or 14-bp deletion and Ins: +2960 or 14-bp insertion. (PDF) [file pone.0212750.s005.pdf]

S5 Table. The linkage disequilibrium results for *HLA-G* 3'-UTR and *MICA* in each group (Ct, CKD, KTN and KTR).

	**Ct (n = 75)**				**95% CI**			
Locus 1	***Locus 2***	**D'**	**LOD**	**r^2**	**Low**	**High**	**Variations in *cis***	
+3010 G>C	*+3142 C>G*	1.000	28.470	0.923	0.930	1.000	*+3010 C* and *+3142 G*	*+3010 G* and *+3142 C*
Ins/Del 14bp	*+3142 C>G*	1.000	15.970	0.616	0.890	1.000	*Ins* 14bp and *+3142 G*	*Del* 14bp and *+3142 C*
Ins/Del 14bp	*+3196 C>G*	0.959	13.470	0.612	0.830	1.000	*Ins* 14bp and *+3196 G*	*Del* 14bp and *+3196 C*
Ins/Del 14bp	*+3010 G>C*	0.885	11.650	0.522	0.730	0.960	*Ins* 14bp and *+3010 C*	*Del* 14bp and *+3010 G*
+3142 C>G	*+3196 C>G*	1.000	9.520	0.410	0.830	1.000	*+3142 G* and *+3196 G*	*+3142 C* and *+3196 C*
+3142 C>G	*+3187 A>G*	1.000	9.110	0.351	0.820	1.000	*+3142 G* and *+3187 A*	*+3142 C* and *+3187 G*
+3010 G>C	*+3187 A>G*	1.000	8.820	0.324	0.810	1.000	*+3010 C* and *+3187 A*	*+3010 G* and *+3187 G*
+3027 C>A	*+3035 C>T*	1.000	7.990	0.574	0.730	1.000	*+3027 C* and *+3035 C*	*+3027 A* and *+3035 T*
MICA-129 Val/Met	*MICA A5.1/Wt*	1.000	6.990	0.265	0.770	1.000	*MICA-129 Met* and *MICA Wt*	*MICA-129 Val* and *MICA A5.1*
+3010 G>C	*+3196 C>G*	0.886	6.910	0.349	0.670	0.960	*+3010 C* and *+3196 G*	*+3010 G* and *+3196 C*
Ins/Del 14bp	*+3187 A>G*	1.000	5.900	0.216	0.730	1.000	*Ins* 14bp and *+3187 A*	*Del* 14bp and *+3187 G*
+3003 C>T	*+3142 C>G*	1.000	4.580	0.202	0.670	1.000	*+3003 C* and *+3142 C*	*+3003 T* and *+3142 G*
+3003 C>T	*+3010 G>C*	1.000	4.080	0.186	0.640	1.000	*+3003 C* and *+3010 G*	*+3003 T* and *+3010 C*
+3187 A>G	*+3196 C>G*	1.000	3.340	0.144	0.570	1.000	*+3187 G* and *+3196 C*	*+3187 A* and *+3196 G*

	**CKD (n = 94)**				**95% CI**			
Locus 1	***Locus 2***	**D'**	**LOD**	**r^2**	**Low**	**High**	**Variations in *cis***	
+3010 G>C	*+3142 C>G*	1.000	39.270	0.958	0.950	1.000	*+3010 C* and *+3142 G*	*+3010 G* and *+3142 C*
+3142 C>G	*+3187 A>G*	0.964	16.860	0.497	0.840	1.000	*+3142 G* and *+3187 A*	*+3142 C* and *+3187 G*
Ins/Del 14bp	*+3142 C>G*	0.965	16.780	0.497	0.840	1.000	*Ins* 14bp and *+3142 G*	*Del* 14bp and *+3142 C*
Ins/Del 14bp	*+3010 G>C*	0.964	15.780	0.474	0.840	1.000	*Ins* 14bp and *+3010 C*	*Del* 14bp and *+3010 G*
+3010 G>C	*+3187 A>G*	0.894	13.900	0.446	0.750	0.960	*+3010 C* and *+3187 A*	*+3010 G* and *+3187 G*
Ins/Del 14bp	*+3196 C>G*	0.862	13.420	0.478	0.720	0.940	*Ins* 14bp and *+3196 G*	*Del* 14bp and *+3196 C*
+3142 C>G	*+3196 C>G*	1.000	11.780	0.344	0.850	1.000	*+3142 G* and *+3196 G*	*+3142 C* and *+3196 C*
+3010 G>C	*+3196 C>G*	1.000	11.140	0.329	0.850	1.000	*+3010 C* and *+3196 G*	*+3010 G* and *+3196 C*
Ins/Del 14bp	*+3187 A>G*	0.943	8.960	0.254	0.750	0.990	*Ins* 14bp and *+3187 A*	*Del* 14bp and *+3187 G*
MICA-129 Val/Met	*MICA A5.1/Wt*	1.000	6.710	0.186	0.760	1.000	*MICA-129 Met* and *MICA Wt*	*MICA-129 Val* and *MICA A5.1*
+3027 C>A	*+3035 C>T*	1.000	6.410	0.319	0.670	1.000	*+3027 C* and *+3035 C*	*+3027 A* and *+3035 T*
Ins/Del 14bp	*+3035 C>T*	0.913	5.700	0.196	0.640	0.980	*Ins* 14bp and *+3035 T*	*Del* 14bp and *+3035 C*
+3187 A>G	*+3196 C>G*	0.913	5.030	0.153	0.630	0.980	*+3187 G* and *+3196 C*	*+3187 A* and *+3196 G*
+3035 C>T	*+3142 C>G*	1.000	4.320	0.125	0.640	1.000	*+3035 C* and *+3142 C*	*+3035 T* and *+3142 G*
+3010 G>C	*+3035 C>T*	1.000	4.090	0.120	0.630	1.000	*+3010 G* and *+3035 C*	*+3010 C* and *+3035 T*
+3003 C>T	*+3010 G>C*	1.000	3.890	0.131	0.610	1.000	*+3003 C* and *+3010 G*	*+3003 T* and *+3010 C*
+3003 C>T	*+3142 C>G*	1.000	3.680	0.125	0.600	1.000	*+3003 C* and *+3142 C*	*+3003 T* and *+3142 G*

	**KTN (n = 36)**				**95% CI**			
Locus 1	***Locus 2***	**D'**	**LOD**	**r^2**	**Low**	**High**	**Variations in *cis***	
+3010 G>C	*+3142 C>G*	1.000	18.860	1.000	0.910	1.000	*+3010 C* and *+3142 G*	*+3010 G* and *+3142 C*
Ins/Del 14bp	*+3010 G>C*	0.911	6.200	0.421	0.660	0.980	*Ins* 14bp and *+3010 C*	*Del* 14bp and *+3010 G*
Ins/Del 14bp	*+3142 C>G*	0.911	6.200	0.421	0.660	0.980	*Ins* 14bp and *+3142 G*	*Del* 14bp and *+3142 C*
+3010 G>C	*+3187 A>G*	0.906	6.070	0.399	0.640	0.980	*+3010 C* and *+3187 A*	*+3010 G* and *+3187 G*
+3142 C>G	*+3187 A>G*	0.906	6.070	0.399	0.640	0.980	*+3142 G* and *+3187 A*	*+3142 C* and *+3187 G*
+3010 G>C	*+3196 C>G*	1.000	5.540	0.372	0.710	1.000	*+3010 C* and *+3196 G*	*+3010 G* and *+3196 C*
+3142 C>G	*+3196 C>G*	1.000	5.540	0.372	0.710	1.000	*+3142 G* and *+3196 G*	*+3142 C* and *+3196 C*
Ins/Del 14bp	*+3196 C>G*	0.672	3.420	0.331	0.390	0.840	*Ins* 14bp and *+3196 G*	*Del* 14bp and *+3196 C*
+3027 C>A	*+3035 C>T*	1.000	3.120	0.412	0.480	1.000	*+3027 C* and *+3035 C*	*+3027 A* and *+3035 T*
Ins/Del 14bp	*+3187 A>G*	0.860	3.030	0.183	0.470	0.960	*Ins* 14bp and *+3187 A*	*Del* 14bp and *+3187 G*
MICA-129 Val/Met	*MICA A5.1/Wt*	1.000	2.430	0.219	0.450	1.000	*MICA-129 Met* and *MICA Wt*	*MICA-129 Val* and *MICA A5.1*

	**KTR (n = 28)**				**95% CI 95**			
Locus 1	***Locus 2***	**D'**	**LOD**	**r^2**	**Low**	**High**	**Variations in *cis***	
+3010 G>C	*+3142 C>G*	1.000	7.900	0.866	0.830	1.000	*+3010 C* and *+3142 G*	*+3010 G* and *+3142 C*
Ins/Del 14bp	*+3196 C>G*	1.000	7.180	0.726	0.780	1.000	*Ins* 14bp and *+3196 G*	*Del* 14bp and *+3196 C*
Ins/Del 14bp	*+3142 C>G*	1.000	5.060	0.559	0.710	1.000	*Ins* 14bp and *+3142 G*	*Del* 14bp and *+3142 C*
+3142 C>G	*+3187 A>G*	1.000	4.540	0.552	0.700	1.000	*+3142 G* and *+3187 A*	*+3142 C* and *+3187 G*
Ins/Del 14bp	*+3010 G>C*	1.000	4.010	0.484	0.660	1.000	*Ins* 14bp and *+3010 C*	*Del* 14bp and *+3010 G*
+3142 C>G	*+3196 C>G*	1.000	3.380	0.406	0.590	1.000	*+3142 G* and *+3196 G*	*+3142 C* and *+3196 C*
MICA-129 Val/Met	*MICA A5.1/Wt*	1.000	2.220	0.188	0.400	1.000	*MICA-129 Met* and *MICA Wt*	*MICA-129 Val* and *MICA A5.1*

Analysis performed in Haploview. The linkage disequilibrium (LD) considered significant had an LOD > 3.000. LOD: is the log of the likelihood odds ratio. 95% CI relative to D': confidence interval. Ct: Control group. CKD: Patients with chronic kidney disease. KTN: Kidney-transplant patients with no rejection. KTR: Kidney-transplant patients who developed episodes of rejection. *Wt*: wild type, which does not show *MICA A5.1* variation. *Del*: +2960 or 14-bp deletion and *Ins*: +2960 or 14-bp insertion.
