# Supplementary material for: The association of HLA-G polymorphisms and the synergistic effect of sMICA and sHLA-G with chronic kidney disease and allograft acceptance
Source: PLoS One. 2019 Feb 22;14(2):e0212750. doi: 10.1371/journal.pone.0212750 (PMC6386361; doi:10.1371/journal.pone.0212750)
Supplement: S6 Table — The inferences of HLA-G haplotype through ELB algorithm of HLA-G alleles with determined UTRs. Analysis performed in Arlequin. The significant linkage disequilibrium had a p < 0.05. Ct: Control group. CKD: Patients with chronic kidney disease. KTN: Kidney-transplant patients with no rejection. KTR: Kidney-transplant patients who developed episodes of rejection. (PDF) [file pone.0212750.s006.pdf]

S6 Table. The linkage disequilibrium results for *HLA-G* 3'-UTR and *MICA* in each group (Ct, CKD, KTN and KTR).

Ct (n = 75)					CKD (n = 94)			
p	**r^2**	**D'**	**UTR**	***HLA-G* alleles**	**UTR**	**D'**	**r^2**	***p***
0.036	0.029	+0.790	UTR-4	*HLA-G*01:01*	---	---	---	---
<0.001	0.282	+0.583	UTR-5	*HLA-G*01:03*	UTR-5	+0.377	0.142	<0.001
<0.001	0.161	+1.000	UTR-13		UTR-13	+0.225	0.033	0.012
0.008	0.046	+0.306	UTR-8		---	---	---	---
<0.001	0.194	+0.440	UTR-3	*HLA-G*01:04*	UTR-3	+0.375	0.134	<0.001
0.003	0.056	+1.000	UTR-15		---	---	---	---
0.01	0.044	+0.731	UTR-1		---	---	---	---
0.009	0.045	+0.533	UTR-2	*HLA-G*01:06*	UTR-10	+0.225	0.033	0.012

KTN (n = 36)					KTR (n = 28)			
p	**r^2**	**D'**	**UTR**	***HLA-G* alleles**	**UTR**	**D'**	**r^2**	***p***
0.04	0.058	+0.760	UTR-1	*HLA-G*01:01*	---	---	---	---
<0.001	0.422	+0.731	UTR-5	*HLA-G*01:03*	UTR-5	+1.000	0.491	<0.001
<0.001	0.189	+1.000	UTR-13		---	---	---	---
<0.001	0.480	+0.813	UTR-3	*HLA-G*01:04*	---	---	---	---
0.032	0.064	+0.294	UTR-10	*HLA-G*01:06*	---	---	---	---

The inferences of *HLA-G* haplotype through ELB algorithm of *HLA-G* alleles with determined UTRs. Analysis performed in Arlequin. The significant linkage disequilibrium had a $p < 0.05$. Ct: Control group. CKD: Patients with chronic kidney disease. KTN: Kidney-transplant patients with no rejection. KTR: Kidney-transplant patients who developed episodes of rejection.
