# Supplementary material for: The association of HLA-G polymorphisms and the synergistic effect of sMICA and sHLA-G with chronic kidney disease and allograft acceptance
Source: PLoS One. 2019 Feb 22;14(2):e0212750. doi: 10.1371/journal.pone.0212750 (PMC6386361; doi:10.1371/journal.pone.0212750)
Supplement: S7 Table — Q1: First Quartile. Q3: Third Quartile. (PDF) [file pone.0212750.s007.pdf]

S7 Table. Association analysis performed between soluble molecules and genetic factors.

sMICA and haplotypes			*MICA-129 Met/ Wt*	*MICA-129 Val/ Wt*	*MICA-129 Val/ A5.1*	
n =	113	n =	23	38	52	
Kruskal-Wallis test =	0.327	Median (pg/mL) =	80.57	87.44	93.02	
Median (pg/mL) =	85.62	Q1-Q3 =	52.90 - 110.73	54.38 - 150.89	63.54 - 156.08	
Q1-Q3 =	56.03 - 141.02	Mann-Whitney U test results	---	0.319	0.140	*MICA-129 Met/ Wt*
			---	---	0.581	*MICA-129 Val/ Wt*
			---	---	---	*MICA-129 Val/ A5.1*

sHLA-G and alleles			*HLA-G*01:01*	*HLA-G*01:03*	*HLA-G*01:04*	
n =	112	n =	85	9	18	
Kruskal-Wallis test =	0.448	Median (ng/mL) =	27.53	23.12	29.14	
Median (ng/mL) =	27.19	Q1-Q3 =	17.84 - 41.93	15.66 - 32.95	18.91 - 50.54	
Q1-Q3 =	17.83 - 42.11	Mann-Whitney U test results	---	0.352	0.468	*HLA-G*01:01*
			---	---	0.208	*HLA-G*01:03*
			---	---	---	*HLA-G*01:04*

sHLA-G and UTRs			UTR-1	UTR-2	UTR-3	UTR-4	UTR-5	
n =	99	n =	30	27	25	9	8	
Kruskal-Wallis test =	0.585	Median (ng/mL) =	27.53	24.29	28.79	20.87	21	
Median (ng/mL) =	26.64	Q1-Q3 =	20.78 - 48.37	15.07 - 46.43	23.46 - 40.58	16.51 - 36.85	17.62 - 28.22	
Q1-Q3 =	17.59 - 42.28	Mann-Whitney U test results	---	0.684	0.630	0.433	0.267	UTR-1
			---	---	0.288	0.827	0.724	UTR-2
			---	---	---	0.274	0.106	UTR-3
			---	---	---	---	0.462	UTR-4
			---	---	---	---	---	UTR-5

Q1: First Quartile. Q3: Third Quartile.
