# Supplementary figures and images for: The association of HLA-G polymorphisms and the synergistic effect of sMICA and sHLA-G with chronic kidney disease and allograft acceptance
Source: PLoS One. 2019 Feb 22;14(2):e0212750. doi: 10.1371/journal.pone.0212750 (PMC6386361; doi:10.1371/journal.pone.0212750)

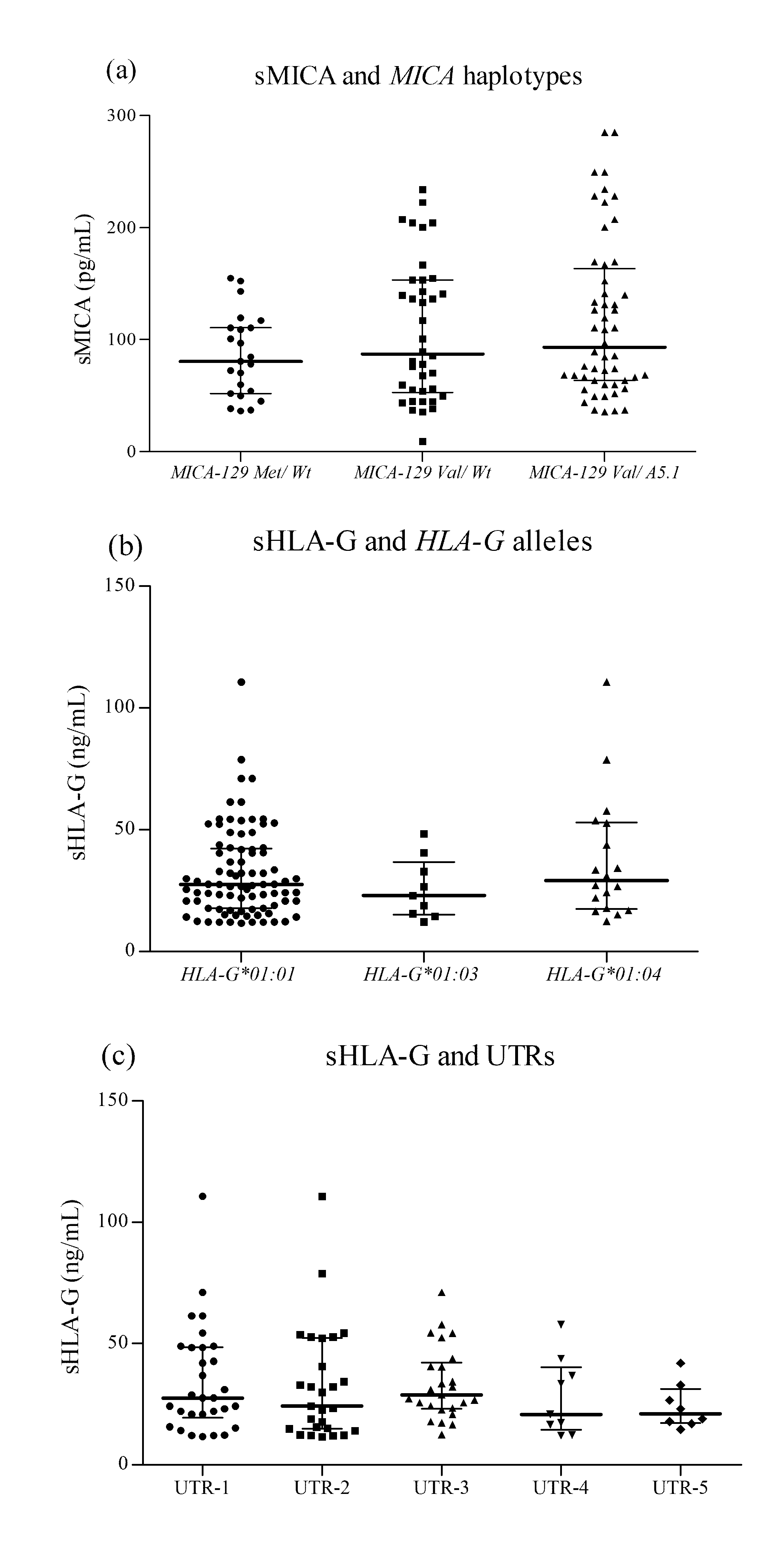

Supplement: S1 Fig — Soluble MICA (sMICA) and MICA haplotypes (MICA-129 Val/Met and MICA A5.1/Wt) association (p = 0.327) (a). Soluble HLA-G and HLA-G alleles association (p = 0.448) (b). Soluble HLA-G and most frequent HLA-G UTRs association (p = 0.585) (c). (TIF) [file pone.0212750.s009.tif]
